# Pyrazole compound BPR1P0034 with potent and selective anti-influenza virus activity

**DOI:** 10.1186/1423-0127-17-13

**Published:** 2010-02-23

**Authors:** Shin-Ru Shih, Tzu-Yun Chu, Gadarla Randheer Reddy, Sung-Nain Tseng, Hsiun-Ling Chen, Wen-Fang Tang, Ming-sian Wu, Jiann-Yih Yeh, Yu-Sheng Chao, John TA Hsu, Hsing-Pang Hsieh, Jim-Tong Horng

**Affiliations:** 1 Department of Medical Biotechnology and Laboratory Science, Chang Gung University, Taoyuan 333, Taiwan; 2 Department of Biochemistry, Chang Gung University, Taoyuan333, Taiwan; 3 Research Center for Emerging Viral Infections, Chang Gung University, Taoyuan 333, Taiwan; 4 Division of Biotechnology and Pharmaceutical Research, National Health Research Institutes, Miaoli 350, Taiwan; 5 Department of Biological Science and Technology, National Chiao Tung University, Hsinchu 300, Taiwan

## Abstract

**Background:**

Influenza viruses are a major cause of morbidity and mortality around the world. More recently, a swine-origin influenza A (H1N1) virus that is spreading via human-to-human transmission has become a serious public concern. Although vaccination is the primary strategy for preventing infections, influenza antiviral drugs play an important role in a comprehensive approach to controlling illness and transmission. In addition, a search for influenza-inhibiting drugs is particularly important in the face of high rate of emergence of influenza strains resistant to several existing influenza antivirals.

**Methods:**

We searched for novel anti-influenza inhibitors using a cell-based neutralization (inhibition of virus-induced cytopathic effect) assay. After screening 20,800 randomly selected compounds from a library from ChemDiv, Inc., we found that BPR1P0034 has sub-micromolar antiviral activity. The compound was resynthesized in five steps by conventional chemical techniques. Lead optimization and a structure-activity analysis were used to improve potency. Time-of-addition assay was performed to target an event in the virus life cycle.

**Results:**

The 50% effective inhibitory concentration (IC_50_) of BPR1P0034 was 0.42 ± 0.11 μM, when measured with a plaque reduction assay. Viral protein and RNA synthesis of A/WSN/33 (H1N1) was inhibited by BPR1P0034 and the virus-induced cytopathic effects were thus significantly reduced. BPR1P0034 exhibited broad inhibition spectrum for influenza viruses but showed no antiviral effect for enteroviruses and echovirus 9. In a time-of-addition assay, in which the compound was added at different stages along the viral replication cycle (such as at adsorption or after adsorption), its antiviral activity was more efficient in cells treated with the test compound between 0 and 2 h, right after viral infection, implying that an early step of viral replication might be the target of the compound. These results suggest that BPR1P0034 targets the virus during viral uncoating or viral RNA importation into the nucleus.

**Conclusions:**

To the best of our knowledge, BPR1P0034 is the first pyrazole-based anti-influenza compound ever identified and characterized from high throughput screening to show potent (sub-μM) antiviral activity. We conclude that BPR1P0034 has potential antiviral activity, which offers an opportunity for the development of a new anti-influenza virus agent.

## Background

**Figure 1:**
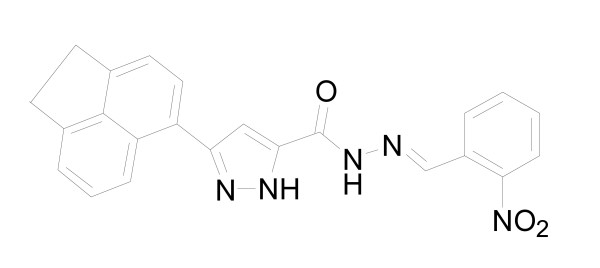
**Chemical structure of BPR1P0034**.

Influenza viruses are respiratory pathogens that affect humans and are responsible for substantial morbidity, mortality, and decreased productivity. Vaccination provides the primary protection from influenza virus infections. Because of the continuous evolution of major viral antigens, vaccine strains must be selected annually. Therefore, vaccine production may not satisfy the need during an influenza pandemic. Antiviral drugs provide a valuable addition to the options available to control influenza infections. Two classes of these antiviral drugs, adamantanes and neuraminidase (NA) inhibitors, are currently prescribed for the prophylaxis and treatment of influenza infections [1]. Adamantanes (amantadine and rimantadine) target the proton channel formed by the viral M2 protein. Because this protein is absent in influenza B viruses, adamantanes have no antiviral effect on this virus type [2]. The rapid spread of resistance to adamantanes in recent years [3,4] has diminished the usefulness of this class of drugs. Two NA inhibitors, orally bioavailable oseltamivir and inhaled zanamivir, are the only drugs currently recommended for the treatment of both influenza A and B virus infections. Mutations identified in the NAs of viruses selected in the presence of NA inhibitors vary depending on the NA antigenic type/subtype and on the drug used [5]. NA is a surface antigen containing an enzymatic active site that is targeted by NA inhibitors. Therefore, viruses with reduced drug susceptibility can emerge as a result of drug use and/or natural genetic variation in NA. A noticeable increase in the emergence of drug-resistant influenza A (H1N1) viruses has been observed in the United States, starting from the end of October of 2007 and spreading widely in 2008. These findings are consistent with reports of the emergence of oseltamivir resistance in influenza A (H1N1) viruses in China, Europe, and other countries [6,7]H. H5N1 viruses resistant to oseltamivir also pose a threat to the public [8-10]. More recently, a swine-origin influenza A (H1N1) virus that is being spread via human-to-human transmission has become a serious public concern around the world [11-14]. A search for influenza-inhibiting drugs is thus particularly important in the face of new pandemic strains and high rate of emergence of influenza strains resistant to several existing influenza antivirals. Cell-based primary screening antiviral assays have the ability to simultaneously screen broad classes of compounds against the functions of multiple viral targets, as well as screen for toxicity [15-17]. The development of a robust and large-scale anti-influenza virus cell-based assay has been established by Noah et al [16]. The objective of this study was to identify novel anti-influenza inhibitors using a similar cell-based neutralization (inhibition of virus-induced cytopathic effect) assay. After screening 20,800 randomly selected compounds from a library, we found that BPR1P0034 has potent inhibitory activity (Fig. 1).

## Methods

### Cell lines, virus strains, and chemicals

Madin-Darby canine kidney (MDCK), Vero, and human rhabdomyosarcoma (RD) cells were maintained in Dulbecco's modified Eagle's medium (DMEM) supplemented with 10% heat-inactivated fetal bovine serum, penicillin (100 U/mL), and streptomycin (100 μg/mL), and the MDCK cells were also fed with additional L-glutamine (2 mM; Gibco) and a nonessential amino acid mixture (0.1 mM; Gibco). Both cell lines were maintained at 37°C in an incubator under 5% CO_2 _in normal air. Influenza virus A/WSN/33 and other strains (Table 1) were propagated in MDCK cells and enteroviruses and echovirus 9 were amplified in RD cells. Amantadine was purchased from Sigma-Aldrich (St Louis, MO, USA) and zanamivir (Relenza) was synthesized and provided by Dr Chun-Cheng Lin (National Tsing-Hua University, Taiwan).

**Table 1:** Antiviral activity of BPR1P0034.

	Concentration (μM)			
	
	BPR1P0034		Amantadine	Zanamivir
		
	IC_50_	CC_50_^c^	IC_50_	IC_50_
Cytotoxic effect				
MDCK		6.75 ± 0.08		
				
Influenza virus				
A/WSN/33 (H1N1)^b^	0.21 ± 0.07		> 25	0.06 ± 0.02
A/Udorn/72 (H3N2)^a^	2.11 ± 0.41		0.98 ± 0.15	0.67 ± 0.23
A/TW/83/05 (H3N2)^a^	1.38 ± 0.08		> 25	0.92 ± 0.03
A/TW/785/05 (H1N1)^a^	0.92 ± 0.11		0.38 ± 0.24	1.06 ± 0.98
B/TW/710/05^b^	8.11 ± 3.98		> 25	0.03 ± 0.02
B/TW/70325/05^b^	1.94 ± 0.72		> 25	0.02 ± 0.01
B/TW/99/07^b^	8.90 ± 4.16		> 25	0.10 ± 0.01
				
Enterovirus 71^b^				
TW/1743/98	> 40	> 20	--	--
TW/4643/98	> 20	> 20	--	--
				
Echovirus 9^b^	> 20	> 20	--	--

MDCK cells or Vero cells were infected with influenza virus and EV71, respectively, and incubated with different concentrations of BPR1P0034.

IC_50_was determined with a plaque reduction assay^a ^or a neutralization assay^b^

^c^CC_50 _was determined with an MTT assay

--, not determined

The values are means ± SD of 2--3 independent experiments.

### Viral titer determination by plaque assay

Monolayer MDCK cells (6 × 10^5 ^cells/well) were washed once with Dulbecco's phosphate-buffered saline (DPBS), and infected with a serially diluted viral suspension. After adsorption for 1 h at 37°C, the viral suspension was replaced with overlay medium, E_0 _(DMEM with penicillin [100 U/mL], streptomycin [100 μg/mL], L-glutamine [2 mM], and nonessential amino acid mixture [0.1 mM]) containing 2.5 μg/mL trypsin and 0.3% agarose. After incubation for 2-3 days at 37°C under 5% CO_2_, the cells were fixed with 10% formaldehyde and then stained with 1% crystal violet. The titer of the virus was expressed in plaque-forming units (PFU) per milliliter.

### Neutralization test and IC_50 _determination

Tissue culture plates (96-well) were seeded with 2.4 × 10^4^cells/well and incubated for 16-20 h at 37°C under 5% CO_2_. The cells were washed once with DPBS, then infected with virus (MOI 3 × 10^-5^), and were overlaid with 50 μL of DMEM containing varying concentrations of test compounds. After incubation at 37°C under 5% CO_2 _for 72 h, the cells were treated with 100 μL of 4% paraformaldehyde for 20 min, and stained with 0.1% crystal violet for 15 min at room temperature. The density of the cells was measured at 570 nm with a VICTOR^3 ^enzyme-linked plate reader (PerkinElmer). The concentration of compound necessary to reduce the virus-induced cytopathic effect (CPE) by 50%, relative to the virus control was expressed as the IC_50_.

### 3-(4,5-Dimethylthiazol-2-yl)-2,5-diphenyltetrazolium bromide (MTT) assay

MDCK cells (2.4 × 10^4 ^cells/well) were seeded in a 96-well plate and incubated at 37°C overnight before the addition of varying concentrations of test compounds. After incubation at 37°C for 72 h, the cells were washed with Hank's balanced salt solution (HBSS; Gibco) and 50 μL/well of MTT solution (5 mg/mL) was added, and then the cells were incubated at 37°C for 3 h. The medium was carefully withdrawn without touching the cells. DMSO (200 μL per well) was added to dissolve the crystal violet, and the absorbance at OD_570 _was read with an ELISA reader. The 50% cytotoxic concentration (CC_50_) of the drug resulted in 50% cell death compared with that in the no-drug control. The CC_50 _was determined according to the Reed-Muench method.

### CPE inhibition test

MDCK cells (2.5 × 10^6 ^cells/well) were seeded in a 6 cm tissue culture dish, grown to confluence, and then challenged with virus (multiplicity of infection [MOI] = 5). After adsorption of virus for 1 h at 37°C, the cells were washed with HBSS, after which E_0 _containing the test compound was added. After incubation for 10 h, the CPE was evaluated under a microscope.

### Plaque reduction assay

MDCK cells (6 × 10^5 ^cells/well) were seeded into six-well tissue culture plates and incubated overnight. The cells were incubated with influenza virus at approximately 50 PFU/well with or without different concentrations of the test compound. After adsorption of the virus for 1 h at 37°C, the viral suspension was removed and the cells were washed with HBSS. The cells were then overlain with E_0 _containing 0.3% agarose with or without the test compound. After incubation for 2-3 days at 37°C under 5% CO_2_, the cells were fixed with 10% formaldehyde and then stained with 1% crystal violet. The numbers of plaques were counted and the percentages (plaque number in the presence of compounds relative to that of untreated control) of antiviral activity were calculated.

### Time-of-addition assay

MDCK cells (6 × 10^5 ^cells/well) were seeded in six-well tissue culture plates and incubated overnight. The cells were challenged with virus (MOI = 5) on ice for 1 h. After adsorption of the virus for 1 h, the viral suspension was removed and the cells were washed with HBSS, then replenished with fresh E_0 _medium (pi = 0). The test medium containing 1 μM of compound was added during the periods -2 to -1 h (preadsorption), -1 to 0 h (adsorption), 0 to 2 h, and 2 to 10 h. After each incubation period, the monolayer was washed with HBSS and incubated with fresh medium until 10 h postinfection (p.i.). The supernatant was collected and the viral yield was determined by plaque assay.

### Western blot

MDCK cells (2 × 10^6^) were seeded in a 6 cm dish and incubated overnight. The cells were infected with influenza A/WSN/33 virus (MOI = 0.5) for 1 h with or without BPR1P0034 (5 μM), and were harvested at various times p.i. The total cell lysate was resolved with sodium dodecyl sulfate-polyacrylamide gel electrophoresis and the proteins were transferred to polyvinylidene difluoride membranes, which were incubated with primary antibody for 1 h and then with the appropriate secondary antibody. The proteins were detected using an enhanced chemiluminescence western blotting detection system (Millipore, Billerica, MA, USA). Rabbit polyclonal antibodies directed against nucleocapsid protein (NP) of influenza A virus or against glyceraldehyde-3-phosphate dehydrogenase (GAPDH) were from Abcam (ab46967) and Santa Cruz Biotechnology (sc-25778), respectively.

### Indirect immunofluorescence assay

MDCK cells (1 × 10^5 ^cells) were seeded on a coverslip and incubated overnight. The cells were infected with influenza virus A/WSN/33 (MOI = 5) for 1 h with or without BPR1P0034 (5 μM) and the coverslips were fixed with 4% paraformaldehyde for 20 min at room temperature at various p.i. time points. The coverslips were incubated with blocking solution (0.5% bovine serum albumin in phosphate-buffered saline) for 1 h at room temperature and then reacted with rabbit anti-NP primary antibody diluted in blocking solution for 1.5 h. After the coverslips had been washed three times with blocking solution, they were incubated with the appropriate Alexa-Fluor-488-labeled secondary antibody and the fluorescence was evaluated with a Zeiss Axiovert 200 M microscope.

### Measurement of Viral RNA Synthesis by Quantitative Reverse Transcription-Polymerase Chain Reaction (Q-RT-PCR)

MDCK cells were infected with A/WSN/33 at an MOI of 5, and total RNA was extracted from cells at various times by using TRIzol (Invitrogen, Carlsbad, CA, USA). The first strand of cDNA was synthesized by using MMLV reverse transcriptase (Invitrogen). Real time Q-RT-PCR was performed in a 20 μl reaction mixture containing 50 nM forward and reverse primers, 1 × SYBR green master mix (Protech Technology Enterprise, Taiwan), and various amounts of template. Fluorescence emitted by SYBR green was detected by using the ABI StepOne Plus sequence detection system (Applied Biosystems, Foster City, USA). To quantify changes in gene expression, the ΔCt method was used to calculate relative changes normalized against the GAPDH gene, as described in User Bulletin No. 2 (Applied Biosystems). The Ct is defined as the cycle at which fluorescence is determined to be significantly greater than background. The ratio of viral RNA to the internal control was normalized to the RNA level at 0 h p.i, which was arbitrarily set to 1. M1 forward primer, 5'GAC CAA TCC TGT CAC CTC; M1 reverse primer, 5'GAT CTC CGT TCC CAT TAA GAG; GAPDH forward primer, 5'AAG AAG GTG GTG AAG CAG GC; GAPDH reverse primer, 5'TCC ACC ACC CTG TTG CTG TA.

***General synthesis of BPR1P series ***(see Additional file 1).

## Results

### BPR1P0034 inhibits influenza virus in a cell-based neutralization assay

Randomly selected 20,800 compounds from a library collection purchased from ChemDiv, Inc., (San Diego, CA, USA, http://www.chemdiv.com) were screened for their capacity to inhibit influenza-virus-induced cell death in a neutralization assay. Influenza virus A/WSN/33 was used and the compounds were tested at a concentration of 10 μM in an assay using MDCK cells, in which GS4071 (oseltamivir carboxylate) was used as the positive control. A reduction in virus-induced cell death indicated antiviral activity. BPR1P0034 (Fig. 1) was one of several initial hits displaying an IC_50 _of less than 10 μM, with no apparent cytotoxicity. The IC_50 _of BPR1P0034 was 0.21 ± 0.07 μM (Table 1). The morphology of the inhibition of influenza-virus-infected CPE in the presence and absence of test compound or virus was evaluated by microscopy. The influenza-virus-infected MDCK cells became rounded and eventually became detached. This CPE was significantly reduced when 1 μM BPR1P was added (data not shown). There was no obvious change in cell morphology when the cell culture medium contained the same concentration of BPR1P.

### Lead optimization

The hit compound was optimized by preparing analogues and further refined by examining their structure-activity relationships (SARs). The lead compound structure was divided into three pharmacophores, R^1^, R^2^, and R^3^, and 34 analogues were prepared for the SAR study (Additional file 2). The activity of these analogues was determined with a cell-based neutralization assay. By varying the hydrophobic part of the pyrazole scaffold in R^1^, we prepared more than 10 analogues, the most potent of which contained the acenaphthene group (BPR1P0034). We varied R^2 ^of the lead compound by changing the hydrazone part, thus preparing 18 analogues. From the assay results, we found that the presence of a nitro group at the ortho position was essential for anti-influenza-virus activity (BPR1P0034). By changing pharmacophore R^3^, we prepared four analogues, three of which showed moderate activity (5 < IC_50 _< 25 μM). We synthesized a total of 34 analogues. Nine of them showed moderate activity and eight showed good activity (IC_50 _< 5 μM). The most active compound synthesized was the lead compound BPR1P0034, which exhibited an IC_50 _of 0.21 ± 0.07 μM in the neutralization assay.

### Mechanism-of-action studies

**Figure 2:**
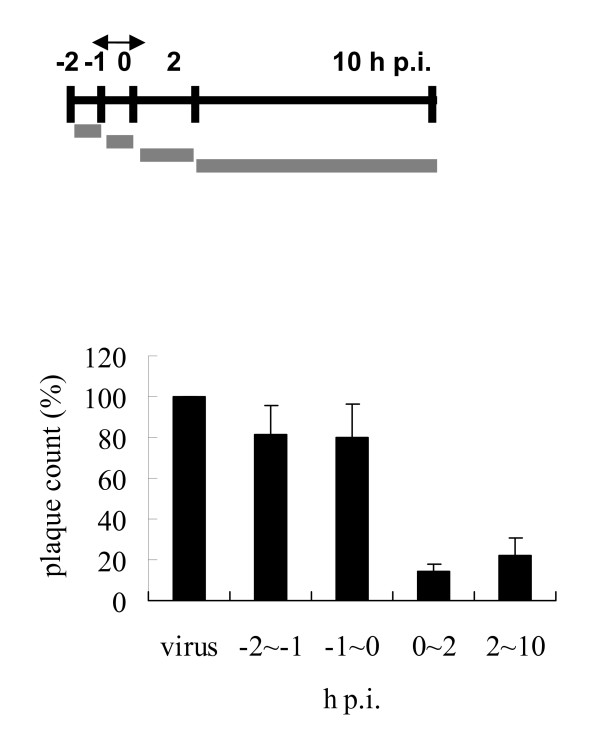
**Time of addition of BPR1P0034**. (A) MDCK cells were inoculated with influenza A/WSN/33 virus at MOI = 5. BPR1P0034 was added at the indicated times. Viral infection was performed between --1 h and 0 h. (B) After each incubation period, the test medium containing 1 μM of compound was removed and the cells were incubated with fresh medium until 10 h postinfection. The supernatant was collected and viral yield was determined by plaque assay. This is a representative result of two independent experiments.

**Figure 3:**
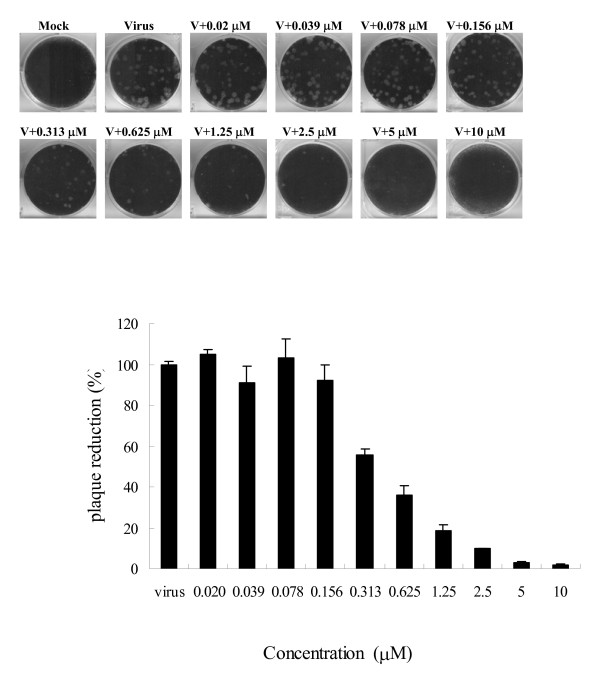
**BPR1P0034 reduced influenza virus production**. MDCK cells were infected with influenza virus A at 50 PFU/well. Different concentrations of BPR1P0034 were added at the stages of viral adsorption and postinfection. (A) The effect of BPR1P0034 on viral plaque formation. (B) Quantification of viral plaques treated with serial dilutions of BPR1P0034. This is a representative result of two independent experiments. The y-axis represents percentage of plaque reduction compared with the virus alone control, set as 100%.

We also examined the broad-spectrum activity of this compound by subjecting it to a bioevaluation study against a variety of strains and viruses, including influenza A and B viruses (four and three strains, respectively, and most of them were recent clinical isolates), enterovirus 71 (two serotypes), and echovirus 9. We used amantadine and zanamivir (Relenza) as positive controls. The results are shown in Table 1. As shown, zanamivir showed excellent activity against both influenza A and B viruses. Amantadine showed no activity against influenza B viruses, as expected. Our compound exhibited similar activity against both A and B viruses demonstrating that it inhibited not only laboratory-adapted WSN strain with long passage history but also several strains isolated after 2005. However, no antiviral activity against enteroviruses or echovirus 9 was observed. To investigate the possible time-dependent inhibitory effects on influenza replication, BPR1P0034 was added to monolayers of infected cells before or after viral adsorption, and the virus yield was assessed using the plaque formation assay (Fig. 2). When the compound was added before viral adsorption (from -2 to 0 h p.i.), no reduction in viral yield was detected. However, BPR1P0034 displayed a significant inhibitory effect on influenza virus when added after viral adsorption, particularly in the early stage (from 0 to 2 h p.i.), suggesting that BPR1P0034 affects the early steps of the replication cycle, such as uncoating or vRNA importation into the nucleus but this requires further evaluation. At this concentration, viral plaque formation was clearly inhibited (Fig. 3).

We tested the inhibitory activity of BPR1P0034 against influenza viral polymerase using a plasmid-based ribonucleoprotein (RNP) assay. In brief, we added BPR1P0034 to HEK293 cells coexpressing influenza virus A/WSN/33 (H1/N1) polymerase complex proteins, including PA, PB1, PB2, and NP, together with the reporter plasmid, pPOLI-CAT-RT. The reporter plasmid contained a vRNA-like RNA encoding the reporter gene for chloramphenicol acetyltransferase (*CAT*), in the negative sense, flanked by the 5' and 3' noncoding regions of the NS vRNA segment of the influenza virus [18]. BPR1P did not inhibit the RNA-dependent RNA polymerase (RdRp) at concentrations of up to 10 μM (data not shown). We conclude that the polymerase of influenza virus A/WSN/33 (H1N1) may not be the primary molecular target of BPR1P0034, based on the cell-based RdRp assay. We also addressed the activity of BPR1P0034 in inhibiting NA using an in vitro neuraminidase inhibition assay [19]. A formaldehyde-inactivated influenza virus suspension from MDCK cells infected with influenza virus was produced and used in the neuraminidase inhibition assay. To evaluate the inhibitory activity of BPR1P0034, inactivated virus supernatant was preincubated with the test compounds for 30 minutes at 30°C. The enzymatic reactions were performed for one hour at 37°C, terminated, and the fluorescence intensity measured using the fluorogenic substrate 2'-(4-methylumbelliferyl)-α-D-N-acetylneuraminic acid [20] with a spectrofluorometer. The viral titer was below the detection limit, with no reduction in NA activity (results not shown). The effects of BPR1P0034 on influenza virus were further evaluated with a plaque reduction assay. BPR1P0034 at various concentrations was evaluated in viral plaque reduction assays for influenza virus A/WSN/33. BPR1P0034 dose dependently reduced the PFU produced by infection of MDCK cells with influenza virus (Fig. 3). The IC_50 _value for viral plaque formation was estimated to be 0.42 ± 0.11 μM. The IC_50 _of GS4071 was approximately 40 nM (not shown).

### Inhibition of viral protein and RNA synthesis

**Figure 4:**
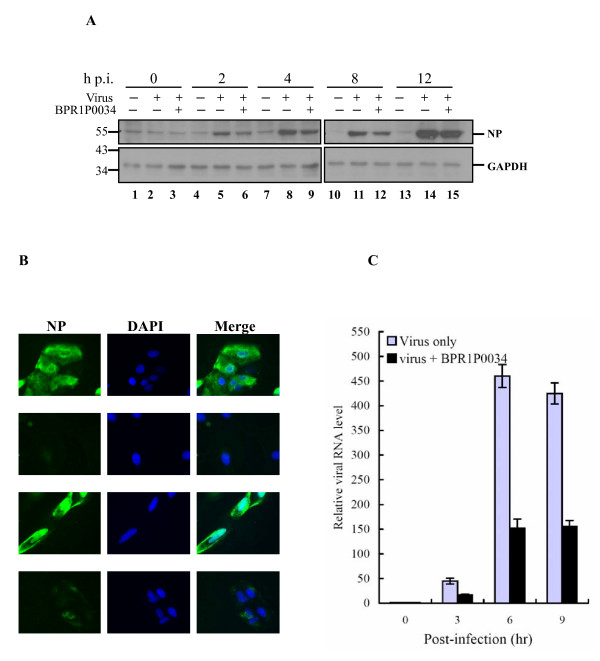
**Inhibition of influenza viral NP protein and RNA synthesis by BPR1P0034**. MDCK cells were infected with influenza virus A/WSN/33 (MOI = 0.5) and incubated with or without 5 μM BPR1P0034 in the adsorption and postinfection stages. The sample was subjected to western blotting (A), immunofluorescence microscopy (B), or quantitative PCR (C). (A) The infected cells were harvested at the times indicated and evaluated by western blotting using an anti-NP antibody. GAPDH was used as the loading control. This is representative of two independent experiments. (B) Influenza A/WSN/33 virus (MOI = 5)-infected MDCK cells on coverslips were incubated with or without BPR1P0034 during viral adsorption and after infection. The cells were fixed for the indicated times and reacted with primary anti-NP antibody and Alexa-Fluor-488-labeled secondary antibody. The production of viral protein, represented by NP, was detected by immunofluorescence microscopy. (Row A) Cells infected with influenza virus and harvested at 3 h p.i; (Row B) The same as (Row A) but in the presence of BPR1P0034; (Row C) Cells infected cells and harvested at 6 h p.i; (Row D) The same as (Row C) but in the presence of BPR1P0034. (C) The MDCK cells were challenged with virus in the presence or absence of BPR1P0034 and total RNA was extracted at indicated times. Equal amounts of total RNA (3 μg) were used for quantitative RT-PCR analysis. To quantify the changes in gene expression, the ΔCt method was used to calculate relative changes which were normalized to the GAPDH gene. The ratio of viral RNA to the internal control was normalized to the RNA level at 0 h p.i, which was arbitrarily set to 1.

In the time-of-addition assay, BPR1P0034 demonstrated the most potent antiviral activity at 0-2 h p.i., when viral replication and translation actively occur (Fig. 2). We then investigated whether BPR1P0034 inhibited viral protein and RNA synthesis in an infectious cycle. In a time course experiment, MDCK cells were infected with influenza virus (MOI = 0.5) and were treated with or without BPR1P0034. Cells were harvested at indicated times p.i. and subjected to western blotting using an antibody specific against NP protein. The viral protein produced by the influenza virus (represented by NP) was evident 2 h p.i., and its expression persisted thereafter. The NP produced by influenza virus (Fig. 4A, lanes 6, 9, 12, and 15) was reduced in BPR1P0034-treated cells compared with that in untreated cells (a mean of 2.06 compared to a mean of 1.29 constituting a difference of 1.60 fold at 8 h p.i. from two independent experiments) (Fig. 4A, lanes 5, 8, 11, and 14). This suggests that the reduction in viral protein production is attributed to the inhibition by BPR1P0034 of viral translation. We used immunofluorescence microscopy to confirm this observation. MDCK cells were infected with influenza virus A/WSN/33 (MOI = 5) and incubated with or without 5 μM BPR1P0034 in the adsorption and postinfection stages. The cells were fixed at 3 or 6 h p.i. and reacted with primary anti-NP antibody and evaluated by immunofluorescence microscopy. NP was distributed not only in the cytoplasm but also in the nuclei of the infected cells (Fig. 4B). The expression of NP was significantly reduced in the presence of BPR1P0034. We also investigated viral RNA synthesis in MDCK cells infected with virus and treated with or without BPR1P0034. Total RNA was extracted and subjected to quantitative PCR analysis in the infected MDCK cells at 3, 6, and 9 hours post adsorption. Viral RNA was significantly reduced by about 55-68% when the cells were treated with compound at a concentration that inhibited virus-induced CPEs (Fig. 4C).

## Discussion

There is an urgent need for new anti-influenza drugs and the objective of this study was to identify novel inhibitors from an in-house database. After screening 20,800 structurally diverse compounds, we found that BPR1P0034 has potent inhibitory activity against influenza virus. This is the first compound ever identified and characterized from high throughput screening possessing sub-micromolar anti-influenza activity [16]. Pyrazole and its derivatives have been developed as antiviral compounds [21-24]. Two new class pyrazole compounds with improved stability were synthesized from aryl diketo acid which was a potent and selective inhibitor against HIV. Zeng et al, reported these compounds exhibited antiviral activity with IC50 below 10 μM [21]. Novel pyrazole compounds were developed via SAR study to inhibit West Nile virus and HSV-1 but the antiviral activity (IC_50_) never reached optimal level (sub-μM concentration) [22-24]. To the best of our knowledge, BPR1P0034 is the only pyrazole compound that possesses the best potent antiviral activity (sub-μM) thus far.

In a mechanistic study, viral yield was reduced by 90% when this compound was added at 0-2 h p.i. in a time-of-addition experiment (Fig. 2). This point represents an early stage of viral infection and many preparatory steps for viral replication come into play at this time, such as viral internalization, uncoating, vRNA importation into the nucleus, and primary transcription and translation. Viral yield was also reduced during incubation with BPR1P0034 at 2-10 h p.i. but less dramatically than during incubation at 0-2 h p.i., although the incubation period was longer. Because viral transcription and translation occur at 2-4 h p.i., we predict that the inhibition of viral proteins before 4 h p.i. will disrupt the expression of late proteins, such as hemagglutinin and NA, involved in packaging and budding. We also hypothesize that BPR1P0034 is a general (nonspecific) inhibitor that inhibits viral infection not only in the early stages but also in the late stages (5-12 h p.i.) of infection, in processes such as RNP export from the nucleus, the glycosylation and transport of viral proteins, NA maturation, and viral packaging. However, we cannot exclude the possibility that BPR1P0034 targets cellular factor(s), provoking a multitude of signaling processes in infected cells that may have key functions in the antiviral response [25]. We tested the inhibitory activity of BPR1P0034 against influenza polymerase in a cell-based assay and found that this compound did not inhibit influenza RNP at concentrations of up to 10 μM, at which concentration plaque formation was completely inhibited (data not shown). The activity of BPR1P0034 in inhibiting NA was tested in an in vitro neuraminidase inhibition assay [19] and it did not inhibit the enzymatic activity of NA at concentrations of up to 40 μM (data not shown). In conclusion, BPR1P0034 is neither a polymerase inhibitor nor an NA inhibitor.

In the time-course experiment, viral NP synthesis was inhibited from as early as 2 h p.i. and remained inhibited for 12 h p.i. (Fig. 4A). The inhibition of viral protein synthesis was further confirmed with immunofluorescence microscopic and quantitative PCR data (Fig. 4B, C). These findings are consistent with the results of the time-of-addition assay, in which BRP1P0034 acted at an early stage of viral infection.

The efficacy of current treatments is limited or uncertain in some populations and situations. For example, oseltamivir has lower clinical effectiveness against influenza B than against influenza A infections in children [26-28]. Moreover, the frequency of viral resistance to M2 and NA inhibitors has increased rapidly among seasonal influenza viruses and is now so widespread that it presents a clinical problem [3,4]. The search for alternative antiviral inhibitors with different target sites has thus become an urgent issue. In this study, we have identified a novel anti-influenza compound BPR1P0034 with an unidentified mode-of-action. It will be interesting to elucidate the underlying mechanism accounting for its antiviral activity. With the established assays [29,30], we tested and found that BPR1P0034 did not inhibit NA or the cell-based viral RNP activity (data not shown). Separate studies will be needed to explore if BPR1P0034 would directly inhibit the functions of virus-encoded proteins such as the M2 ion channel. Very recently, a number of host factors were shown needed for influenza virus replication in host cells [31-33]. Inhibition of influenza virus could be achieved through RNAi knock-down or pharmacological inhibition of the essential host factors. Thus, it is also possible that BPR1P0034 is able to interfere with these essential host functions that are needed for the replication of influenza virus inside the cells. Overall, BPR1P0034 may serve as a starting point for the development of a novel class of anti-influenza virus drugs.

## Competing interests

The authors declare that they have no competing interests.

## Authors' contributions

TYC performed antiviral activity assay, time of addition, plaque reduction, western blotting and immunofluorescence microscopy. GRR and JYY synthesized the compounds. SNT and HLC carried out the compound screening and IC_50 _determination of derivatives. MSW participated in statistical analysis of western blotting data. WFT performed qPCR. JTH, HPH, JTAH, SRS and YSC conceived the study and participated in its design and coordination. JTH and HPH wrote the manuscript. All authors read and approved the final manuscript.

## Supplementary Material

Additional file 1 **Supplementary materials and methods**. General synthesis of BPR1P series-reagents and conditions. Click here for file

Additional file 2 **Supplementary table**. Lead optimization by SAR study. Click here for file
