# Supplementary material for: Pyrazole compound BPR1P0034 with potent and selective anti-influenza virus activity
Source: J Biomed Sci. 2010 Feb 23;17(1):13. doi: 10.1186/1423-0127-17-13 (PMC2838761; doi:10.1186/1423-0127-17-13)
Supplement: Additional file 1 — Supplementary materials and methods. General synthesis of BPR1P series-reagents and conditions. [file 1423-0127-17-13-S1.DOC]

**Additional file 1**

**Supplemental materials and methods**

**General Synthesis of BPR1P Series – BPR1P0034 as an example**

**Reagents and conditions:** a) Methyl magnesium bromide, THF, 0°C–room temp, 2 h; b) Pyridinium dichromate, CH_2_Cl_2_, room temp, 2 h; c) Diethyl glycol, sodium ethoxide, ethanol, room temp, overnight; d) Hydrazine hydrate, ethanol, reflux, 3 h; e) o-Nitrobenzaldehyde, toluene.

**EXPERIMENTAL SECTION**

**1-Acenaphthen-5-yl-ethanol^1^** (**2**)**:** Commercially available acenaphthaldehyde (**1**, 2.0 g, 10.9 mmol) in THF (20 mL) was cooled to 0°C and methyl magnesium bromide (6.52 g, 55.0 mmol) was added dropwise. The reaction mixture was allowed to come to room temperature and was stirred for 2 h. A saturated NH_4_Cl solution (10 mL) was slowly added to hydrolyze the adduct at 0°C and the mixture was stirred for 10 min. The phases were separated and the aqueous layer was extracted with ethyl acetate. The combined organic layers were washed with brine, dried over Na_2_SO_4_, and filtered. The filtrate was evaporated under vacuum and the residue was purified by chromatography to produce the desired alcohol **2**. ^1^H NMR (CDCl_3_) δ 1.67 (d, 3H), 1.94 (s, 1H), 3.36 (d, 4H), 5.55 (q, 1H), 7.27 (d, 1H), 7.28 (d, 1H), 7.45 (t, 1H), 7.56 (d, 1H), 7.81 (d, 1H).

**1-Acenaphthen-5-yl-ethanone** (**3**)**:** Pyridinium dichromate (3.0 g, 9.0 mmol) was added to a stirred solution of alcohol **2** (1.8 g, 9.0 mmol) and powdered 4Å molecular sieve (0.75 g) in anhydrous CH_2_Cl_2_ (50 mL) at 0°C. After the addition was complete, the mixture was stirred at room temperature for 2 h, then diluted with anhydrous ether and filtered through celite. The filtrate was concentrated under vacuum and the residue was purified by chromatography to yield ketone **3**. ^1^H NMR (CDCl_3_) δ 2.73 (s, 3H), 3.42 (s, 4H), 7.29 (d, 1H), 7.35 (d, 1H), 7.57 (t, 1H), 8.06 (d, 1H), 8.71 (d, 1H).

**4-Acenaphthen-5-yl-2,4-dioxo-butyric acid ethyl ester^2^** (**4**)**:** Diethyl oxalate (1.0 g, 6.9 mmol) was added dropwise to a suspension of sodium ethoxide (0.5 g, 6.9 mmol) in 20 mL of ethanol at 8–10°C, followed by the dropwise addition of ketone **3** (1.4 g, 4.7 mmol) in 30 min. The reaction mixture was stirred overnight at room temperature. The reaction mixture was quenched with HCl (10 mL, 6 M), then extracted with ethyl acetate, washed with brine, dried over sodium sulfate, and evaporated. The residue was purified by chromatography to produce ester **4**. ^1^H NMR (CDCl_3_) δ 1.39 (t, 3H), 3.44 (s, 4H), 4.37 (q, 2H), 7.12 (s, 1H), 7.34 (d, 1H), 7.39 (d, 1H), 7.59 (t, 1H), 8.04 (d, 1H), 8.54 (d, 1H).

**5-Acenaphthen-5-yl-2H-pyrazole-3-carboxylic acid hydrazide^3^** (**5**)**:** To ester **4** (1.2 g, 4.3 mmol) in ethanol (10 mL) was added hydrazine hydrate (0.6 g, 9 mmol). The solution was refluxed for 2 h, then filtered. The reaction mixture was washed with ethanol to produce pure hydrazide **5**. ^1^H NMR (DMSO-d_6_) δ 3.81 (s, 4H), 4.47 (s, 2H), 7.11 (s, 1H), 7.37 (d, 1H), 7.39 (d, 1H), 7.51 (t, 1H), 7.63 (d, 1H), 8.22 (d, 1H), 9.66 (s, 1H), 13.61 (s, 1H).

**5-Acenaphthen-5-yl-2H-pyrazole-3-carboxylic acid (2-nitro-benzylidene)-hydrazide** (**6, BPR1P034**)**:** Hydrazide **5** (0.5 g, 1.2 mmol) and o-nitrophenyl carbaxaldehyde (0.19 g, 1.3 mmol) were dissolved in toluene and refluxed overnight on Dean Stark apparatus. The reaction mixture was filtered and washed with CH_2_Cl_2_ and methanol to produce the pure final product **6**. ^1^H NMR (DMSO-d_6_) δ 3.40 (s, 4H), 7.12 (s, 1H), 7.37–8.15 (m, 6H), 8.96 (s, 1H), 12.24 (s, 1H), 13.84 (s, 1H).

**References**

1. Huang, W. S., Hu, Q.-S., Pu, L., 1999. From Highly Enantioselective Monomeric Catalysts to Highly Enantioselective Polymeric Catalysts:  Application of Rigid and Sterically Regular Chiral Binaphthyl Polymers to the Asymmetric Synthesis of Chiral Secondary Alcohols. J. Org. Chem. 64, 7940-56.

1. Fadnavis, N. W., Radhika, K. R., 2004. Enantio- and regiospecific reduction of ethyl 4-phenyl-2,4-dioxobutyrate with baker’s yeast: preparation of (*R*)-HPB ester, Tetrahedron Asymmetry 15:3443-7.
2. Zhang, J., Didierlaurent, S., Fortin, M., Lefrancois, D., Uridat, E., Vevert, J.P., 2000. Potent nonpeptide endothelin antagonists: synthesis and structure-activity relationships of pyrazole-5-carboxylic acids. Bioorg. Med. Chem. Lett. 10, 2575-8.
