# Supplementary material for: Pyrazole compound BPR1P0034 with potent and selective anti-influenza virus activity
Source: J Biomed Sci. 2010 Feb 23;17(1):13. doi: 10.1186/1423-0127-17-13 (PMC2838761; doi:10.1186/1423-0127-17-13)
Supplement: Additional file 2 — Supplementary table. Lead optimization by SAR study. [file 1423-0127-17-13-S2.DOC]

**Additional file 2**

**Supplementary Table**

Optimization of R^1^

| **BPR Code** | **Structure** | **IC_50_ (μM)** | **CC_50_ (μM)** | **SI**^a^ |
| --- | --- | --- | --- | --- |
| BPR1P0033S0 |  | 1.36 ± 0.63 | > 12.5 | > 9.19 |
| BPR1P0034S0 |  | 0.21 ± 0.07 | > 12.5 | > 59.52 |
| BPR1P0046S0 |  | > 25 | > 25 | - |
| BPR1P0047S0 |  | 2.66 ± 0.45 | 7.04 ± 0.036 | 2.65 |
| BPR1P0049S0 |  | 1.10 ± 0.58 | 15.22 ± 2.83 | 13.84 |
| BPR1P0050S0 |  | > 25 | > 25 | - |
| BPR1P0051S0 |  | > 25 | > 25 | - |
| BPR1P0052S0 |  | 6.17 | 20.34 ± 2.91 | 3.30 |
| BPR1P0054S0 |  | > 25 | > 25 | - |
| BPR1P0055S0 |  | 1.10 ± 0.58 | > 25 | > 22.72 |
| BPR1P0058S0 |  | 14.68 ± 1.60 | > 25 | > 1.70 |
| BPR1P0060S0 |  | 2.83 ± 0.35 | 6.22 ± 0.44 | 2.20 |
| BPR1P0061S0 |  | 0.98 ± 0.21 | 10.33 ± 0.08 | 10.54 |

Optimization of R^2^

| **BPR Code** | **Structure** | **IC_50_ (μM)** | **CC_50_ (μM)** | **SI**^a^ |
| --- | --- | --- | --- | --- |
| BPR1P0034S0 |  | 0.21 ± 0.07 | > 12.5 | > 59.52 |
| BPR1P0035S0 |  | > 25 | 1.21 ± 0.23 | - |
| BPR1P0036S0 |  | > 25 | > 25 | - |
| BPR1P0037S0 |  | > 25 | > 25 | - |
| BPR1P0038S0 |  | > 25 | > 25 | - |
| BPR1P0039S0 |  | > 25 | > 25 | - |
| BPR1P0040S0 |  | > 25 | > 25 | - |
| BPR1P0041S0 |  | > 25 | 21.24 ± 0.3 | - |
| BPR1P0042S0 |  | > 17.9 | > 25 | - |
| BPR1P0043S0 |  | > 25 | > 25 | - |
| BPR1P0044S0 |  | > 25 | > 25 | - |
| BPR1P0063S0 |  | 17.79 | > 25 | > 1.41 |
| BPR1P0064S0 |  | 10.44 ± 0.07 | > 25 | > 2.39 |
| BPR1P0065S0 |  | 8.38 ± 0.57 | > 25 | > 2.98 |
| BPR1P0066S0 |  | > 25 | > 25 | - |
| BPR1P0067S0 |  | > 25 | > 25 | - |
| BPR1P0068S0 |  | > 25 | > 25 | - |
| BPR1P0069S0 |  | > 25 | > 25 | - |

Optimization of R^3^

| **BPR Code** | **Structure** | **IC_50_ (μM)** | **CC_50_ (μM)** | **SI**^a^ |
| --- | --- | --- | --- | --- |
| BPR1P0053S0 |  | > 25 | > 25 | - |
| BPR1P0056S0 |  | 17.25 ± 3.07 | > 25 | > 1.45 |
| BPR1P0057S0 |  | 14.93 ± 7.31 | > 25 | > 1.67 |
| BPR1P0059S0 |  | 15.04 ± 1.11 | > 25 | > 1.66 |

^a^SI (selectivity index) is the ratio of CC50 to IC50
